# Say that again: Quantifying patterns of production for children with autism using recurrence analysis

**DOI:** 10.3389/fpsyg.2022.999396

**Published:** 2022-10-21

**Authors:** Amanda Mankovich, Jessica Blume, Kacie Wittke, Ann M. Mastergeorge, Alexandra Paxton, Letitia R. Naigles

**Affiliations:** ^1^Department of Psychological Sciences, UConn Child Language Lab, University of Connecticut, Storrs, CT, United States; ^2^Human Development and Family Sciences, Research in Early Developmental Studies Lab, Texas Tech University, Lubbock, TX, United States; ^3^Department of Speech, Language, and Hearing Sciences, University of Connecticut, Storrs, CT, United States; ^4^Department of Psychological Sciences, University of Connecticut, Storrs, CT, United States; ^5^Center for the Ecological Study of Perception and Action, University of Connecticut, Storrs, CT, United States

**Keywords:** autism, grammar, productivity, language development, recurrence

## Abstract

The current research study characterized syntactic productivity across a range of 5-year-old children with autism and explored the degree to which this productivity was associated with standardized measures of language and autism symptomatology. Natural language samples were transcribed from play-based interactions between a clinician and participants with an autism diagnosis. Speech samples were parsed for grammatical morphemes and were used to generate measures of MLU and total number of utterances. We applied categorical recurrence quantification analysis, a technique used to quantify patterns of repetition in behaviors, to the children’s noun-related and verb-related speech. Recurrence metrics captured the degree to which children repeated specific lexical/grammatical units (i.e., recurrence rate) and the degree to which children repeated combinations of lexical/grammatical units (i.e., percent determinism). Findings indicated that beyond capturing patterns shown in traditional linguistic analysis, recurrence can reveal differences in the speech productions of children with autism spectrum disorder at the lexical and grammatical levels. We also found that the degree of repeating noun-related units and grammatical units was related to MLU and ADOS Severity Score, while the degree of repeating unit combinations (e.g., saying “the big fluffy dog” or the determiner-adjective-adjective-noun construction multiple times), in general, was only related to MLU.

## Introduction

In this paper, we use “children with autism” or “children with ASD” as part of a choice to focus on person-first language with this specific developmental sample. We recognize that adults in the autistic community have increasingly advocated for identity-first language (Vivanti, 2020), but this preference has not yet been investigated or established in children. Albeit outside the scope of the current work, we encourage future researchers to investigate preferences for identity- versus person-first language in children so that scholars and others in the field can honor the needs of this community.

The development of grammar marks a shift from the ability to construct relatively simple sentences (e.g., “want ball”) to the ability to express more complex ideas (e.g., “I want the large green ball”). Interestingly, compared to typically developing peers, many studies have reported that children with autism spectrum disorder (ASD) exhibit a much wider range of spoken language abilities, including their acquisition and use of grammar (see Eigsti et al., 2011, for review). Variation in production across the spectrum has been demonstrated through measurements of utterance length (e.g., mean length of utterance, or MLU), utterance complexity (e.g., grammatical morphemes and clauses), and amount of word-/utterance-level repetition of a social partner (e.g., echolalia; Kjelgaard and Tager-Flusberg, 2001).

Researchers have proposed that variations in language production are based on *why* children with ASD communicate (Chevallier et al., 2012; Yoder et al., 2015; Mundy et al., 2019; Su et al., 2021). Children with ASD may communicate selectively because of their varying levels of social motivations, such as whether or not they are intentional in their communication and if they are, how varied their pragmatic functions are (e.g., requesting, seeking information, liking, social maintaining, and social orienting). Consistent with this idea, the *elicited bootstrapping hypothesis*, an extension of the transactional model of language development, has suggested that these differences in social motivations may activate a chain reaction with consequences for language production (Camarata and Yoder, 2002; Sameroff, 2009; Su et al., 2021). That is, reduced motivations within social contexts may suppress interest in and production of communication bids. Fewer attempts to communicate thereby provide fewer opportunities to elicit and absorb communicative responses, limiting children’s access to functional language models, which may also reduce how much the child speaks.

This variability in social interest to communicate likely contributes to a broad range of language production profiles observed among children with ASD. For instance, if a child is unmotivated to talk within a social interaction, they may say very little to their communication partners, or they may only communicate for a restricted range of pragmatic functions, such as to request (e.g., “*I want bear*”). Additionally, they may use a frozen phrase such as “I want _____,” rarely using that same pronoun “I” with other verbs. Such restricted and repetitive production profiles make it challenging to assess whether the child’s language knowledge is abstract (e.g., manifesting subject-verb-object structure), and whether their language use is productive or creative. Producing additional utterances within the turn, such as “We bought the toys yesterday” or “I like cuddly animals at the zoo,” points to both abstract and productive usage, but requires more talk and hence more motivation to talk.

Linguists have commonly referred to the ability to creatively combine units of meaning (morphology) into complex structures (syntax) as *productivity* (see Baker, 1979; Pinker, 1989; Tomasello, 2000; Hoff, 2012). It is not immediately clear what the wide range of spoken language levels across only a few contexts implies for productivity in ASD. Understanding productivity is critical: Productivity can have trickle-down effects on other components of language, impacting communicative competence (Yorio, 1980; Pinker, 1989; Tomasello, 2000). For instance, children with more frequent and varied productions may later develop a broader vocabulary, which enables them to talk about a wider range of topics. A better grasp of how early grammar manifests productivity among children with autism may help therapists select the most effective targets in clinical sessions.

The objective of the current study is to quantify indicators of productivity across a range of verbal children with ASD and to characterize how these children might vary in their productivity. We introduce a new method of characterizing productivity—namely, recurrence analysis, a nonlinear time series analysis technique used across several disciplines to capture underlying structural patterns of the system (Leonardi, 2012; Webber and Marwan, 2015). Because recurrence analysis involves continuous measurements, it may be particularly well-suited in order to precisely and accurately capture the variability in children’s language productivity across the autism spectrum.

### Variability in the grammar of children with ASD

Recent work has focused on exploring the nature of structural language production in autism, specifically syntax and morphology (e.g., Park et al., 2012; Zhou et al., 2015; see Boucher, 2012, for review). Of particular interest has been whether children with ASD have typically developing morphological and syntactic language use. Compared with typically developing children (either age-matched or language-matched), the development of syntax and morphology in speech is frequently protracted for children with ASD (Bartolucci et al., 1980; Howlin, 1984; Eigsti et al., 2007; Park et al., 2012; Zhou et al., 2015; Brynskov et al., 2017; Chin et al., 2018; see Boucher, 2012, for review). This line of work suggests that children with ASD produce less complex speech than matched TD children, often measured by *mean length of utterance* (MLU), which counts the morphemes a child uses in their utterances.

In one study, Eigsti et al. (2007) recorded language samples during free play from 5-year-olds with ASD and from TD children matched on vocabulary, talkativeness, and non-verbal mental age. Compared to TD children, children with ASD produced utterances that were less syntactically complex (i.e., containing fewer verb phrases, noun phrases, and sentence structures), and shorter (i.e., smaller MLU). Thus, these children with ASD appeared to experience syntactic delays separate from lexical achievements.

A longitudinal study by Tek et al. (2014) found both similar and different patterns to Eigsti et al. (2007) cross-sectional data. Across 24 months of development, one ASD subgroup (32 months old at study onset) showed slower growth in MLU and total number of utterances compared to a TD group matched on expressive language skills (20 months old at study onset). This ASD group also lagged on the production of several specific grammatical elements, including a range of verb types and markers plus noun plurals. In contrast, another ASD subgroup developed grammar at similar rates to the TD group (see also Bartolucci et al., 1980; Howlin, 1984; Park et al., 2012).

Thus, more recent work suggests that not all children with ASD follow the same language acquisition trajectories (Kjelgaard and Tager-Flusberg, 2001; Modyanova et al., 2017; Wittke et al., 2017; see Naigles and Chin, 2015, for review). For instance, when using standardized assessments, Kjelgaard and Tager-Flusberg (2001) found at least three language-related subgroups of children with ASD, including those with language impairment, who exhibited language difficulties across all tested syntactic and semantic domains, those with borderline language deficits, who exhibited fewer language difficulties across tested syntactic and semantic domains, and those with neurotypical language. This work marked a call to characterize the entire spectrum of language abilities in ASD, particularly beyond just vocabulary size.

More recent work has continued to compare grammar use in subgroups of children with ASD. For example, Modyanova et al. (2017) examined subject-verb agreement in the elicited productions of 3- to 16-year-old children with ASD possessing normal language (ALN) and those with language impairment (ALI). Those in the ALI group performed more poorly on their elicited production of the present, past regular, and past irregular tenses compared to the ALN group. However, some children with ASD in both ALI and ALN groups performed similarly to TD children, providing further evidence of variability across the spectrum. Moreover, Wittke et al. (2017) characterized sub-phenotypes for grammatical abilities in the speech of 5-year-olds with ASD who engaged in semi-structured play activities. Their analysis focused on children’s usage of Brown’s (1973) 14 grammatical morphemes, and described three subgroups for the verbal children in their sample: One whose children were highly talkative and virtually error-free in grammatical usage, one whose children were highly talkative but produced numerous grammatical errors, and one whose children produced both fewer and shorter utterances, but whose utterances were relatively error-free.

Taken together, these studies on the heterogeneity of language production in ASD suggest that traditional language sample descriptors like MLU and total utterances do not capture language heterogeneity in describing patterns of typical versus slow and/or grammatically impaired language trajectories, thus warranting more dynamic grammatical analysis strategies. Moreover, in order to understand *productivity* in this population, we will argue that it is important to think about the degree to which children combine new grammatical structures independently from the degree to which they combine words, and keep in mind that MLU conflates word and grammatical unit combinations. Furthermore, the context and topics of the samples contribute to variability in grammatical usage (Kover et al., 2014). As we describe below, studies investigating productivity in children with ASD have yielded mixed results, in part because the measures of productivity have not clearly distinguished word combinations from grammatical combinations.

### Assessments of productivity in ASD compared to TD^1^

Among TD individuals, productivity is usually demonstrated when a person uses a grammatical construction (a) with five or more lexical items (Rispoli et al., 2009), (b) with novel lexical items (Pinker, 1989; Akhtar and Tomasello, 1997), (c) with different morphological endings (Akhtar and Tomasello, 1997; Tomasello et al., 1997), and/or (d) consistently across obligatory contexts (Brown, 1973). In contrast to studies of TD children, which have yielded estimates of consistent productivity by the age of 2 years, examinations of productivity in speech among preschool-aged children with ASD are very limited and have yielded mixed results. That is, some children are found to be consistently productive across grammatical constructions, whereas others show productivity with some constructions but not others (see Roberts et al., 2004; Eigsti et al., 2007; Park et al., 2012; Chin et al., 2018; Le Normand et al., 2018). For instance, Roberts et al. (2004) found no distinguishable differences in the degree to which 5- to 15-year-olds with ASD and language-matched TD children produced past and present tense markers for familiar verbs across obligatory contexts. Similarly, Le Normand et al. (2018) recorded child productions during a narrative-elicitation task and found that the ASD group consisting of 5-year-old French speakers did not differ from the age-matched TD group in their production of verbs, pronouns, the imperfect tense, past participle, and case markers across obligatory contexts. However, their ASD group did produce significantly fewer nouns, adjectives, determiners, and prepositions, meaning that they appeared less productive on these measures. Le Normand and colleagues suggested that nominal morphology may be more difficult for children with ASD to master than verbal morphology.

Furthermore, Eigsti et al. (2007) found that their 5-year-old autism group used significantly fewer subject-verb-object sequences/sentences with three or more different verbs, showing less advanced productivity than their TD group, whereas Park et al. (2012) reported less productivity in preschool-aged children with autism’s spontaneous usage of plurals, “ing,” and 3rd person singular “-s,” but were as productive as the TD group in the usage of articles, auxiliary verbs, and copula verbs. Interestingly, Park et al. (2012) also assessed productivity *via* elicited production of the past tense and plural and found that children whose elicited production of the past tense was not productive nonetheless used the past tense productively in their spontaneous speech.

Additional mixed findings come from a data-rich case study by Chin et al. (2018). Using a Speechome Recorder to collect longitudinal home-based language samples, a 3-year-old child who was later diagnosed with autism was found to produce language comparable to a 2-year-old TD child (matched on language complexity across all the visits) in the number of different verbs they used with each tense/aspect, indicating more advanced productivity. However, compared to the TD child the child with autism produced conventional past, present, and future tenses with fewer verbs and less consistently across obligatory contexts (i.e., less advanced productivity). In other words, the child with autism showed the ability to use grammatical morphemes related to verb tense/aspect but did not do so as flexibly as the TD peer.

Taken together, these findings highlight that establishing the level of productivity manifested by children with ASD in their speech is difficult. Previous studies have primarily examined two types of measures to assess productivity: elicited production scores, from semi-structured procedures meant to elicit specific morphemes, and measures of spontaneous speech from naturalistic language samples. However, elicited production tasks may not be ideal for revealing productivity in children with ASD, because these tasks rely on good participation and social attention. For example, many elicitation tasks provide children with 1–2 stimulus images and prompt children to produce a one-word response using open-ended questions (e.g., “Tell me what he did to the leaves?”) or cloze procedure scaffolding (e.g., “What happened? The boy….[raked]”). Children may also be prompted to produce contrasting morpheme markers using learned non-words that correspond to paired stimulus images (e.g., “How many are there? [one/two wug/wugz]”). Lack of productivity within these tasks, then, could arise because the children do not understand the tasks and so do not provide the correct words, or sometimes even any words, for productivity to be assessed (Boucher, 2012).

Beyond these specific procedures in a research context, we know that measuring language in autism comes with challenges (Tager-Flusberg, 2000). Children with autism often present with differences in social behaviors (e.g., differences in levels of attention in structured tasks) and atypical language behaviors like delayed or immediate echolalia (i.e., the delayed or immediate repetition of a social partner’s utterances; Tager-Flusberg and Calkins, 1990; American Psychiatric Association, 2013). Differences in attention, motivations to communicate, and test-taking skills may make it challenging to elicit long, rich productions in structured contexts, such as during standardized language testing or even semi-structured language interactions (Scarborough et al., 1991; Koegel et al., 1997; Condouris et al., 2003; Su et al., 2021; see Boucher, 2012, for review). For instance, if a clinician tries to elicit a narrative language sample where a child shares a personal story or retells a story from a book, but that child is not interested in the topic, they may produce less language than they might with another topic. And, even if they did produce some language, we might not expect it to be as productive in length, content, and grammatical structures as they would be in the context involving the topic that interested them. In other words, language samples derived from a less engaging context may not be as representative of linguistic skill. As indicated by Kover et al. (2014), the ADOS may offer a more appropriate language sampling context since it comprises several activities, varying across modules and sessions (e.g., Module 2 includes a birthday party task and a snack, whereas Module 3 does not). However, Kover et al. (2014) also point out that the context and speech partners also contribute to children’s proclivity to use a wide range of grammatical devices. Park et al. (2012) suggested that differences in procedures (i.e., semi-structured play versus free play versus elicitation tasks) could account for discrepancies in results between their research and other research. Thus, an approach to production data across a range of activities that potentially taps into varied interests would therefore be critical if we want to characterize children with ASD’s full range of abilities.

Another limitation of productivity studies lies in their statistical approaches. Although they report a large degree of variability in performance during productivity assessments (e.g., Kjelgaard and Tager-Flusberg, 2001; Park et al., 2012; Tek et al., 2014), results have been based on aggregate mean scores (i.e., counts of morphemes and words). Mean scores likely mask interesting patterns of behavior by eliding important variability, and measures of production beyond frequency may provide insights into differences in productivity for these children (Hoff, 2006; see also Müller-Frommeyer et al., 2020).

### Gaps in the literature

While language differences within ASD have been broadly characterized within the literature, several key open questions still exist. First, language development studies of children with ASD have largely focused on group-level differences between children with ASD and age-matched TD peers. However, ASD exists on a spectrum of language abilities that range from minor to severe. The vast range of possible language production outcomes for ASD has not yet been thoroughly investigated.

Second, although we know that lexical and grammatical production abilities range from average to highly impaired, what these differences in language abilities mean for the productivity of syntax—or the degree to which specific lexical and grammatical items are used with different items—remains unclear. For example, we might expect a child who is not productive to only use the word “the” with the word “cat,” whereas a child who is productive would use “the” with all sorts of nouns. Degrees of productivity could be indicated by different recurrence measures, in that children who may be less recurrent in their individual lexical and grammatical productions may also be more recurrent in their *patterns* of productions. For instance, if a child just produces noun phrases (e.g., “the cat,” “a big bear,” and “the bank”), they are highly recurrent in individual grammatical productions (e.g., repeating determiner-noun or determiner-adjective-noun) but less recurrent across a range of grammatical phrases. Having a more advanced syntax means that the child is moving beyond noun phrases; that is, a productive speaker would link noun phrases using verb phrases (e.g., “*would love to* play with the cat”) and prepositional phrases (e.g., “I would love to play with the cat *in the* morning”). This type of analysis is considerably more sensitive than a gross language measure like MLU, which captures the length of utterances but not the grammatical complexity or novelty of word combinations.

Finally, approaches to these group-level differences have been based on composite scores from either standardized tests, lab-based paradigms, or spontaneous speech measures. These measures have been compared using traditional methods of analysis (e.g., means and ranges). However, these methods of analysis make key assumptions about the degree to which different activities elicit the same types of talk. For instance, traditional analyses would suggest that a child who produces rich talk in one task but less advanced talk across several other tasks is relatively unproductive. These analyses are unable to capture data that seem complex or irregular (i.e., children alter speech by task) but may actually involve predictable underlying structures. These analyses are thus problematic given differences in social motivations to talk in autistic individuals (see Chevallier et al., 2012) and the context-sensitivity of language production even among TD individuals (Müller-Frommeyer et al., 2020). These traditional methods also make assumptions about the nature of syntactic abilities within ASD and how components of a linguistic system interact. For instance, earlier analyses of grammatical abilities and productivity have not captured the relative sequential occurrence of recurrent words and grammatical units. That is, currently, it is unclear how individual items (i.e., words and grammatical units) unfold relative to one another across a whole language transcript. This is problematic since the ordering of particular words and grammatical units is essential to understanding the nature of the productivity of syntax.

A nonlinear approach to studying productivity would allow for the representation of linear interactions within child language as well as a broad range of other special component interactions informative to syntax that often get masked by summative analyses. Furthermore, this approach does not make assumptions about the distribution of data points across a sequence or even their stationarity (i.e., how the mean state changes across a sequence of behaviors); this is meaningful for small data sets, as well as data sets that contain outliers. This is true of many language studies containing heterogeneous groups of children with ASD. Thus, one potentially valuable tool to characterize the unfolding of grammatical abilities in ASD into a fruitful syntax typology is RQA, a technique to understand how units of speech repeat across stretches of transcriptions.

### Microlevel assessment of language production

Many studies have focused on standardized testing and language production scores to characterize children’s early language abilities. Furthermore, most assessments of linguistic repetition are not measured quantitatively so degrees of repetition are not really known. An informative alternative to characterizing language abilities would be a more microlevel assessment of children’s productions with a fine-grained analysis of their actual linguistic and grammatical structures—and more specifically, how frequently and in what ways these structures are being repeated. Understanding the nature of repetitions of words and grammar is important because it may provide insights into the degree to which children combine meanings of units in a creative way (i.e., productivity). For example, children who are repetitive in their word combinations (i.e., saying the same words in the same order), perhaps due to delayed echolalia, are likely less productive than children who utter repetitions of grammatical combinations.

Recurrence quantification analysis (RQA) is a nonlinear approach that quantifies change in a system over time (see Marwan et al., 2007; Webber and Marwan, 2015). RQA allows researchers to quantify how a time series repeats values or patterns of values across a period of observation to provide insights into the relative deterministic properties and flow of changes of the target phenomenon (e.g., types of words and grammatical units). While a comprehensive description of RQA is beyond the scope of the current work, we provide a conceptual overview of its principles and procedures; further methodological details and empirical applications can be found in Riley and Van Orden (2005), Orsucci et al. (2006), Coco and Dale (2014), and Leonardi (2012).

Categorical RQA is a variant of RQA that specifically examines the structures and patterns within discrete data, such as language (e.g., Dale and Spivey, 2006). In general, recurrence—or *repetition*—between adult interlocutors has been considered “good” at the pragmatic level because it indicates that the interlocutors are aligned in semantic interests and thereby engaged in the same conversation (i.e., semantic alignment; Dale and Spivey, 2006; Fusaroli et al., 2020, Unpublished manuscript)^2^). However, completely verbatim repetition of the addressee’s previous speech would be considered less ideal since it would not further the dialog, or could reflect the echolalia that may be a reflection of restricted behavior/interests. Thus, recurrence could be inflated by echolalia or perseveration. Recurrence of *specific* patterns, though, could reflect the rehearsal of newly acquired structures with the implied goal of morpheme mastery in functional social communication contexts.

To date, the research comparing grammar and word recurrence has been limited (see Leonardi, 2012, for review). Previous researchers using RQA have focused on (1) lexical mirroring of two TD interlocutors (Dale and Spivey, 2006) and (2) the changes in language styles (i.e., broad function word category items such as pronouns, articles, prepositions, auxiliary verbs, adverbs, conjunctions, and negations) of a single TD interlocutor (Müller-Frommeyer et al., 2020). For instance, in an analysis of recurrence in language styles, Müller-Frommeyer and colleagues found that recurrence rate (i.e., the degree of repetition; RR) was perfectly correlated with the proportion of function words, indicating that our RQA-based approach is meaningful when compared against more traditional metrics. However, compared to monologues, conversations elicited a higher determinism of function words (i.e., a measure of how structured repetitions are across speech). Findings indicate that metrics such as determinism can shed light on the patterning of language which cannot be captured by counts and proportions (i.e., recurrence rate).

We suggest that at the *grammatical* and *word* levels, high recurrence of individual items is indicative of less advanced grammatical and lexical production abilities because the child would simply be repeating themselves. For example, a highly lexically recurrent child might say “the ball” three times without adding in any further details about its size, shape, and capabilities (i.e., to be thrown and bounced). Such lexical repetitions may signify echolalic speech. A highly grammatically recurrent child might reuse the same parts of speech over and over again (i.e., determiner-noun; “the cat,” “a bag,” “my toy”). This latter child might be expected to be less productive as well, as they are not trying out a variety of grammatical units. However, other recurrence parameters focusing on the patterning of words (e.g., percent determinism; %DET) capture something more than simply word count or proportions, including features of the communicative context (e.g., having a conversational partner changed the structure of how function words were used in Müller-Frommeyer et al., 2020). Regular structure in how these items pattern (i.e., %DET) could be indicative of adapting language style to another person across the course of a conversation. This adaptiveness might therefore provide evidence of more advanced grammatical and lexical abilities because the child is practicing new ways to combine units.

Applying RQA to understanding language heterogeneity in autism would address three important gaps in various literatures. From a measurement perspective, assessments of language abilities do not currently respect the continuous nature of the phenomena: Most productivity and repetitive speech measures are currently all-or-none, despite our understanding that autistic language exists on a spectrum. From a methodological perspective, although scholars have claimed that RQA can uncover some structural differences in language, studies have not yet directly compared the grammatical and word levels of analysis. From a language development perspective, researchers have yet to explore the sequential structures that make up noun and verb phrases at both the lexical and grammatical levels. Understanding how repetitive language patterns are structured within these types of phrases has implications for how spoken language production is assessed and described in this population. In summary, RQA has been used to assess diversity and alignment of semantic and lexical productions primarily within typically developing populations. Thus, tackling these topics *via* RQA will add valuable information to understanding the nature of early productivity in ASD.

### Current study

The primary goal of this research is to more subtly characterize the language production of a heterogeneous sample of children with ASD.

We do this first by focusing on the degree to which lexical and grammatical units repeat within the language data from 5-year-old children with ASD. To answer this question, we reanalyze the dataset from Wittke et al. (2017) due to the heterogeneity of syntactic ability within its sample (including, e.g., children who were highly talkative or minimally talkative, children who produced many or few grammatical errors; see Wittke et al., 2017, for additional information about participants and tasks). These data provide an excellent opportunity to apply RQA to capture this variability because the summative analyses used in the initial study may have masked meaningful language information in the sample. Because learning the structure of grammar involves learning how to combine both words and grammatical elements (e.g., nouns, verbs, morphemes) in rule-governed ways, we quantify the degree to which children repeat *specific lexical items* (and *the grammatical units that make up these lexical items*) with items they have never heard in combination before, what we call “syntactic recurrence.”

Second, although Wittke et al. (2017) previously assigned the children to three subgroups based on their NVIQ and percent of grammatical errors, the present analyses do not focus on these subgroups. Instead, we focus on individual differences in the production of phrasal constructions across this sample. At the micro (individual) level, we explore whether repetitions are indicative of language measures that Wittke et al. (2017) calculated from the language samples (e.g., mean length of utterance and total number of utterances).

Third, we investigate these questions by using nonlinear methods (i.e., RQA) to quantify patterns of repetition across an individual child’s speech. Within this type of analysis, each word in the child’s transcript is a sequential datum. Each lexical item is isolated in the transcript and is then divided into morphological and syntactic units. We specifically focus on noun phrases since this grammatical form class develops the earliest (Gentner, 1982; Goldfield and Renick, 1990; Fenson et al., 1994). We also focus on verb phrases since they are crucial pieces for children to start building their very first sentences (Gleitman, 1990; Bloom, 1993).

The current study involved several hypotheses about the mappings between RQA and linguistic structure, not necessarily specific to ASD. Broadly speaking, we test whether more advanced syntax, measured *via* traditional linguistic measures and then *via* RQA, could be an indicator that a child is more productive (i.e., less recurrent). In particular, we hypothesized that producing more utterances overall would be associated with a lower RR, but also with longer sequences (i.e., higher %DET), of repeated units. We also predicted that more complex language (i.e., higher MLU) would be associated with less repetition (lower RR), and with longer sequences at the lexical and grammatical levels of noun and verb phrases (higher %DET).

## Materials and methods

### Corpus

The participant dataset for the current study started with the 189 children with ASD from the Autism Phenome Project (APP). The APP is a longitudinal project conducted at the Medical Investigation of Neurodevelopmental Disorders (MIND) Institute (University of California, Davis), and it examines the neurobiological, genetic, and behavioral features of autism. Children were recruited within northern California with exclusionary criteria based on diagnosis, age, and language exposure (i.e., children were only exposed to English or to both English and Spanish). The first time the children participated in the APP was at age 3 years (Wave 1), often following the child’s initial diagnosis of ASD. However, almost 100 children returned for additional assessments through the APP around 5 years of age (Wave 3; *n* = 98).

Child participants of the APP at Wave 3 engaged in extensive behavioral testing, including standardized language assessments. The comprehensive assessment battery included the *Autism Diagnostic Observation Schedule* (ADOS; Lord et al., 2000), for confirmation of autism diagnostic status; the *Differential Ability Scale*, *Second Edition* (DAS-II; Elliott, 2007), to obtain a non-verbal IQ score; and the *Peabody Picture Vocabulary Test*, *Third Edition* (PPVT-3; Dunn and Dunn, 1997) and *Expressive One-Word Picture Vocabulary Test*, *Third Edition* (EOWPVT-3; Brownell, 2000), to assess both receptive and expressive vocabulary abilities. Previously, Wittke et al. (2017) classified the children based on their language and non-verbal IQ scores (see Table 1). Classifications included: (1) *High Verbal* children, scoring in the typical range (standard scores of 85 and above) for both non-verbal and vocabulary language testing; (2) *Low Verbal* children, whose non-verbal IQ standard scores ranged from 71 to 85 and with standardized testing commensurate with their non-verbal IQ; and (3) *Minimally Verbal* children, whose non-verbal IQ and vocabulary performance was significantly below average (i.e., standard scores of 70 or less). Here, we treat ASD symptomatology and language as continuous variables in order to take advantage of increased variance in the data and to identify patterns with further nuance in the dataset, but a previous analysis of this dataset grouped participants into discrete categories. We present their descriptive statistics in Table 1 to provide an overview of the dataset.

**Table 1:** Means for original groups based on standardized test scores.

Measure	High verbal (*n* = 38)	Low verbal (*n* = 11)	Minimally verbal (*n* = 33)
	M (SD)	M (SD)	M (SD)
Age (in months)	68.63 (12.21)	66.20 (7.60)	68.88 (12.52)
NVIQ	102.95 (11.68)	78.70 (2.54)	56.29^a^ (10.22)
ADOS	11.89 (5.13)	17.80 (4.92)	22.25 (2.76)
DAS verbal	48.44 (8.80)	32.90 (9.35)	13.81^b^ (6.52)
PPVT-3	98.47 (14.33)	75.63 (17.77)	44.67 (10.56)
EOWPVT-3	101.03 (16.52)	76.00 (12.63)	60.94 (7.86)

NVIQ, standard score on differential ability scale, second edition (DAS-II); ADOS, Autism Diagnostic Observation Schedule; DAS verbal = T-score on DAS-II; PPVT-3, standard score on Peabody Picture Vocabulary Test, third edition; EOWPVT-3, standard score on Expressive One-Word Picture Vocabulary Test, third edition. This table is drawn from Wittke et al. (2017).

a Only 14 participants in the *Minimally Verbal* group were able to participate in the *DAS-II* testing. The remainder of this group completed the *Mullen Scale of Early Learning* (MSEL; Mullen, 1995) at Wave 3, and their mean group *T*-scores on this measure was 20 (*SD* = 0), indicating floor-level performance for those children who completed the MSEL.

b This reflects the group mean for only the 14 participants in this group who participated in the DAS-II.

All children were autistic and were diagnosed based on the DSM-IV American Psychiatric Association (2000). Additional exclusion criteria were applied for the current study after screening assessment performance within the available data sample. One child was excluded because autism diagnostic criteria were not met based on ADOS cutoff scores at Wave 3. Another child was excluded because performance on expressive language and speech production measures were affected by intelligibility difficulties exacerbated by suspected childhood apraxia of speech. Furthermore, because the focal research question in the current study concerned language production, an additional 29 children were excluded because they did not produce enough language (i.e., at least 20 utterances) during the ADOS, which was used for retrospectively transcribing spontaneous language samples. This is perhaps unsurprising given that all of these children had also been classified as Minimally Verbal, although two participants from the Minimally Verbal group were included in the sample since they did produce spontaneous language, *N*(utterances) = 33 and 124. Video recordings were not available for an additional 16 children due to recording errors (i.e., session not taped or file corrupted), and so they were also excluded from this analysis.

The final sample comprised 51 of the original 98 children (Wave 3 of the study; *M*_age_ = 68.84, *SD* = 12.77), all of whom had language transcriptions collected from ADOS recordings. The sample included 36 males, 13 females, and 2 children whose sex was not reported. The sample is predominantly male, consistent with evidence that the rate of diagnosis is higher in males and consistent with the growing consensus that females are likely under-diagnosed due to differences in ASD symptomatology that are not well-captured by current assessment tools (Kanner, 1943; Asperger, 1944; Fombonne, 2009; Kreiser and White, 2014). Descriptive statistics for this broader sample can be found in Table 2.

**Table 2:** Sample means for standardized testing and spontaneous speech measures.

Measure	M (SD)	Min	Max
Age (ADOS)	68.84 (12.77)	54	112
NVIQ	95.45 (17.17)	48	146
ADOS (SA)	9.15 (2.10)	3	17
ADOS (RRB)	4.41 (2.26)	0	8
ADOS (Total)	13.57 (5.77)	4	24
ADOS severity score	6.69 (2.10)	2	10
Length of transcript (in minutes)	17.67 (4.22)	10.5	28.5
Total utterances	126.47 (55.39)	29	263
MLU	4.30 (1.62	1.9	9.14
% Ungrammatical utterances	0.08 (0.06)	0	0.29
% Echolalic utterances	0.03 (0.06)	0	0.29
TTR	0.37 (0.11)	0.2	0.67
Verb token	91.27 (53.33)	10	213
Verb type	31.25 (15.53)	5	65
Verb TTR	0.39 (0.11)	0.25	0.69
Noun token	83.59 (47.42)	14	178
Noun type	45.51 (23.43)	9	88
Noun TTR	0.58 (0.11)	0.35	0.85

NVIQ, standard score on differential ability scale, second edition (DAS-II); ADOS, Autism Diagnostic Observation Schedule. MLU, Mean Length of Utterance. TTR, Type-Token Ratio.

### Transcriptions

As stated, recordings of previous behavioral testing were used for collecting language transcripts for this sample. Children engaged with investigators, administrators, and parents in semi-structured tasks from the ADOS that afforded high levels of spontaneous and unprompted language production (Tager-Flusberg et al., 2009). ADOS tasks were generally administered in the standardized order for each Module, although the clinicians occasionally administered tasks out of order when the child’s participation required a change in task type to increase motivation and engagement. Whether the tasks were administered in the standardized sequence or out of the order, all the assigned tasks for these language samples were still transcribed. Of the children in our sample, 25 completed ADOS Module 2, 25 completed ADOS Module 3, and only one completed ADOS Module 1. Language production samples were derived from these tasks and used to construct participants’ grammatical profiles. Language-transcribed tasks varied slightly by ADOS Module administered but generally included: Free Play, Birthday Party, Bubble Play, Snack, Make-Believe Play, Conversation, Description of a Picture, Telling a Story from a Book, Cartoons, and Creating a Story. Although a previous study found that the ADOS yielded less complex and productive language from children with ASD than a parent–child play sample (Kover et al., 2014), those researchers included only the first 15 min of the ADOS for their language sample. We aimed to maximize the potential for language output by including selective tasks that encourage language rather than press for social responses only. All audiotapes were transcribed word-for-word by the third author and an undergraduate research assistant. Audiotapes were listened to multiple times and transcribed verbatim. If an utterance or its parts could not be identified after three passes, it was marked as unintelligible. Transcription reliability was reached *via* a consensus process where transcribers watched video recordings together and checked for differences in codes or errors (Shriberg et al., 1984). All discrepancies were discussed by the transcription team until at least 90% inter-rater agreement (range of 92–98%) was achieved; if line agreement was unable to be achieved, such utterances were consequently coded as unintelligible.

Each utterance was then assigned to a speaker—the child, the parent, or the administrator—but only children’s utterances are included in the current analysis to focus on their individual language use. Given that we were not interested in how much children were repeating others (i.e., echolalia), rather our focus was on how much children were repeating themselves, we included all speech that was produced in our analyses. All transcripts were analyzed using the Computerized Language Analysis (CLAN) software in CHAT format (MacWhinney, 2008). This software takes the words from a text file and categorizes them according to their free and bound morphemes for a categorical analysis at the morpheme level.

### Coding

Our purpose was to analyze both the lexical and grammatical levels of children’s speech production using RQA. As stated earlier, because nouns are one of the first lexical items that children produce, we analyzed the elements of noun phrases. Furthermore, because verbs are necessary to form meaningful sentences, we also analyzed many elements of verb phrases. Thus, language transcriptions were specifically annotated for noun phrase or verb phrase, lexical and grammatical, components (see Tables 3, 4). In addition, to further distinguish noun and verb coding, we did not include any of the noun phrase structures in the verb phrase-related lexical and grammatical coding (see lines 1, 3, 4, and 5 in Table 4). Hence, verb coding is more properly called verb-related rather than verb phrase. CLAN conventions were used to mark morphological aspects of speech transcriptions and syntactic errors.

**Table 3:** Noun-related category codes.

Sub-level of analysis	Grammatical category	Lexical examples
Syntax	1. Common Noun	dog, spoon, book
	2. Proper Noun	Tuesday, Polar Express, Dora
	3. Pronoun	it, that, those, her, I, him, itself, yourself
	4. Determiner	the, a, an, that, those
	5. Adjective	pretty, old, nice, funny
	6. Adverb	over, next, once, about, today, just, all
	7. Gerund	flying, fishing, jumping, swimming
	8. Wh-question	who, what, when, where, why, how
	9. Number	six, seven, eight
	10. Preposition	(go) in (the house), (look) at (the dinosaur)
Morphology	11. Plural	-s, -es, children
	12. Possessive	-‘s, -his, her, my, your, their, our
	13. Superlative	worst, best
	14. Comparative	better, older

The coded items within children’s noun -related syntactic and morphology constructions. All morphology items were double coded as nouns (e.g., plural and possessive) or adjectives (e.g., superlative and comparative). The order of these items was preserved across transcriptions. All non-noun-related syntactic and morphology items (including adjectives and plurals outside of the noun phrase-related syntactic and morphology constructions) were coded as random number sequences.

**Table 4:** Verb-related category codes.

Sub-level of analysis	Grammatical category	Lexical examples
Syntax	1. Verb	go, see, play, want
	2. Adverb	(what’s gonna happen) now
	3. Preposition	(make) up (a story)
	4. Negative	not
	5. Infinitive	to
Morphology	6. 1st/3rd person singular	was
	7. 1st person singular	are, am
	8. 3rd person singular	is, does, wants
	9. Present participle	gonna, doing, destroying
	10. Past participle	stuck, broke, seen
	11. Present tense	are
	12. Past tense	got, dropped, went, did
	13. Modal/Conditional	would, does, can
	14. Progressive	(what else is happen)-ing
	15. Copula	(here it) is
	16. Auxiliary	(what) is (he doing)

The coded items within children’s verb-related syntactic and morphology constructions. All morphology items were double coded as verbs. The order of these items was preserved across transcriptions. All non-verb-related syntactic and morphology items (including adjectives and plurals outside of the verb-related syntactic and morphology construction) were coded as random number sequences and therefore not included in the current analyses. The nouns within the verb-related constructions were also coded as random number sequences (e.g., the word “them” in “go get them” would be coded as a random number), and again not included in the current analyses.

To provide a richer picture of the dataset, we provide an example of the coding below. In this example, a child is responding to a prompt about make-believe play with action figures and tools. Of particular interest is the child’s raw speech: Blue text represents all noun phrase-related components, orange text represents all verb-related components, and black text represents components not involved in noun phrases or verb-related constructions. Again, notice that when the child says, “*you knock it you get more power that way*,” the words “*you*,” “*it*,” and “*more power that way*” are marked as parts of noun phrases for the noun-related coding (in blue). By contrast, the verb-related construction coding (in orange) is largely based on morphology and ignores the nouns entirely.

#### Example 1

**Table:** 

Lexical:	**He can jump** super high.
Grammatical:	**Pronoun**-**modal**-**verb**-1-2.
Lexical:	Higher **than you can fly**.
Grammatical:	3-**preposition**-**pronoun**-**modal**-**verb**.
Lexical:	**Pretend that**’**s just a baseball** and **you can find it to get more power**.
Grammatical:	**Verb**-**pronoun**-**3rd person**-**adverb**-**determiner**-**noun**-4-**pronoun**-**modal**-**verb**-**pronoun**-**infinitive**-**verb**-**noun**-**noun**.
Lexical:	**Watch you can hold this up** and **how** long **you knock it you get more power that way**.
Grammatical:	**Verb**-**pronoun**-**modal**-**verb**-**pronoun**-**adverb**-5-wh**pronoun**-6-**pronoun**-**verb**-**pronoun**-**pronoun**-**verb**-**adverb**-**noun**-**determiner**-**noun**.

All raw text was then converted to numerically identified categories (e.g., all nouns coded as “‘1,” all pronouns coded as “2”). This coding was critical for RQA to reveal how children reuse noun- and verb-related lexical and grammatical structures. To prevent RQA from capturing repeating patterns of non-target grammatical structures, items identified as not being part of noun-related or verb-related lexicon/grammar were coded as unique (i.e., non-repeating) values; this ensured that RQA could only “see” the patterns of language that we were interested in studying here. The coded words within each sentence were strung together in a way that maintained the temporal order of the speech.

### Categorical recurrence quantification analysis

In the current work, we apply RQA to the coded transcripts of child language to examine how patterns of children’s noun- and verb-related phrases change over time. Thus, this new application involves characterizing the lexical and grammatical constructions of the noun and verb-related phrases within a child’s “series” of speech, in which each word in the child’s transcript is a sequential measurement (*cf*. Dale and Spivey, 2006). This is the focus we apply here. That is, we characterize the degree to which an *individual* child repeats specific lexical/grammatical items alone and in combination with items they have never repeated in combination before. For instance, in one example of lexical repetition across noun phrases, a child said:

#### Example 2

**Table:** 

Lexical:	The frog starts going like (unintelligible word).
Grammatical:	**Determiner**-**noun**-**verb**-**thirdpersonsingular**-**verb**-**presentparticiple**-**preposition**.
Lexical:	The frogs are gonna invade the city.
Grammatical:	**Determiner**-**noun**-**plural**-**auxiliary**-**present**-**verb**-**presentparticiple**-**verb**-**determiner**-**noun**.
Lexical:	The frogs are leaving trying to invade the city.
Grammatical:	**Determiner**-**noun**-**plural**-**auxiliary**-**present**-**verb**-**presentparticiple**-**verb**-**presentparticiple**-1-**verb**-**determiner**-**noun**.

Notice the repetitions of “the frog” and “the city” across the utterances. Furthermore, the child is consistently using determiners with their nouns to form noun phrases. In contrast, another child said:

#### Example 3

**Table:** 

Lexical:	Look a frog mom!
Grammatical:	**Verb**-**determiner**-**noun**-**noun**.
Lexical:	They’re flying.
Grammatical:	**Pronoun**-**present**-**verb**-**presentparticiple**.
Lexical:	Hey mom look at frogs are doing.
Grammatical:	1-**noun**-**verb**-**preposition**-**noun**-**plural**-**auxiliary**-**present**-**verb**-**presentparticiple**.

Notice that this second child produces noun phrases with much less repetition both in their lexical items (i.e., “a frog mom,” “they,” and “frogs” referring to the same concepts) and grammatical items (i.e., determiner, adjective, pronoun, and noun).

As shown in these examples, we identify repetitions in individual categories and across sequences of categories—here, words and grammatical units. By comparing these data, we can characterize how the trajectories of word sequences and grammatical constructions might be more vs. less consistent (i.e., frequently vs. infrequently repeated) within a single speech sample.

A strength of using RQA to quantify patterns across an individual child’s speech transcript is that it can be used to examine very short or very long time-series data without assuming a normal distribution of the data (Carello and Moreno, 2005). Although transcriptions varied in the amount of time the children participated in each activity and the number of utterances produced, we decided not to cut longer transcriptions short because these differences in language production are interesting for understanding the wide range of language abilities of children with ASD.

We conducted RQA on the lexical and grammatical data for each child’s transcription using the “crqa” package (*version 1.0.9*; Coco and Dale, 2014) from R in RStudio (*version 1.1.423*; R Core Team, 2021). First, we constructed a recurrence matrix that indicates when a time series returned to a given state (e.g., word repetitions across a transcription). Given that we conducted categorical RQA based on the type of data available, this recurrence matrix included only *exact* repetitions of the categorical state under consideration (e.g., each specific lexical item) across the *entire* time series, even lagged across time (similar conceptually to autocorrelation). A separate recurrence matrix was created for each noun- and verb-related lexical and grammatical time series for each child, resulting in four matrices per child.

As a technical point, calculating recurrence matrices from categorical data requires the researcher to provide a unique categorical identifier for each item of interest so that the recurrence matrix will identify any repetition of the same values in the time series. However, if a researcher wishes to remove data from consideration—say, if items in a specific class are not of interest to the given research question—the researcher must be sure to code the data accordingly: If all items outside of the class of interest are given the same categorical identifier, those not-of-interest items will appear as repetitions in the recurrence matrix, skewing the later steps. In the present study, we were exclusively interested in noun-related and verb-related lexical and grammatical items, so all other items in other classes were given random categorical identifiers (i.e., non-repeating negative numbers) to be sure they were not considered as moments of recurrence in the analysis.

#### Visualization

**Figure 1:**
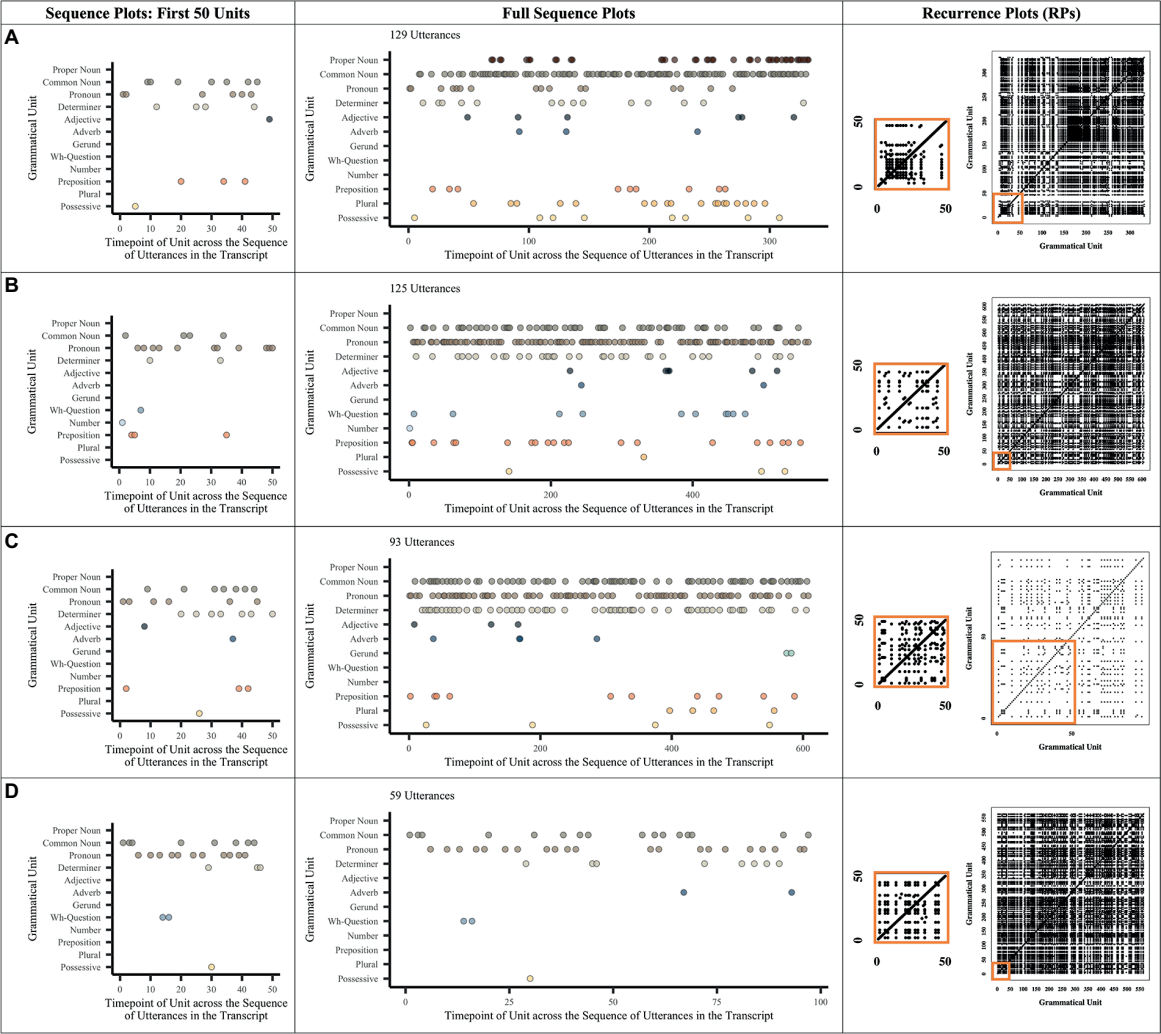
Example Plots for the Noun-related Grammar Production of Four Children. The space not covered by dots in the sequence graph represents instances when a child did not either use one of the noun-related grammatical units listed or produced other units not in the noun phrase (e.g., verb-related units, coordinators, and adjectives). Recall that RR is based on a percentage, not on counts. Child **(A)** produced noun-related speech high in %DET and high in RR. Child **(B)** produced high %DET but low RR. Child **(C)** produced low %DET but higher RR. Child **(D)** produced low %DET and low RR. Looking at children **(A)** and **(D)**, each who produced a similar number of utterances, we see that A has a denser RP and more lines than **(D)**.

**Figure 2:**
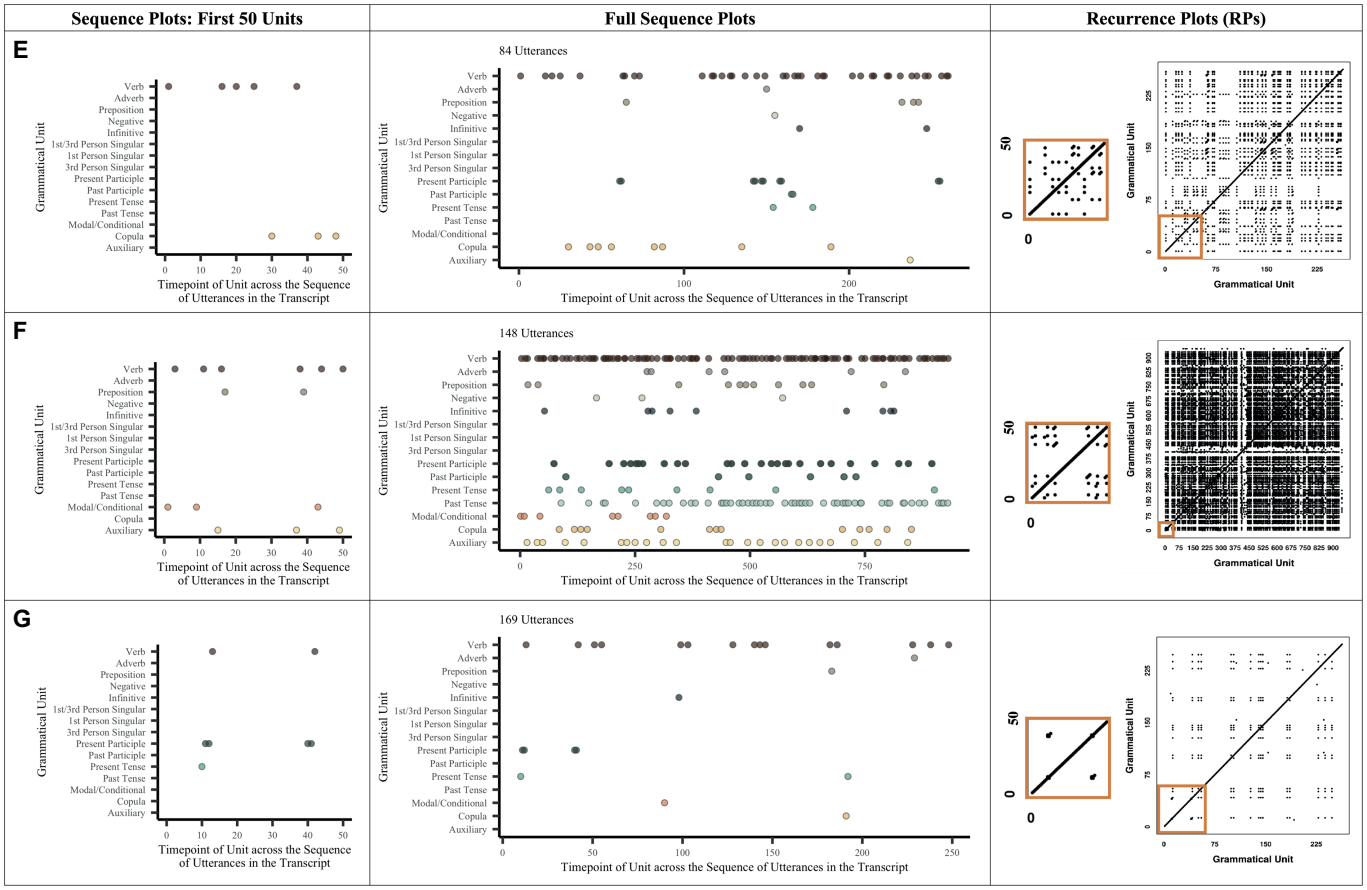
Example Plots for the Verb-related Grammar Production of Three Children. The space not covered by dots in the sequence graph represents instances when a child did not either use one of the verb-related grammatical units listed or produced other units not in the verb phrase (e.g., noun-related units, coordinators, and prepositions). Recall that RR is based on a percentage, not on counts. No child produced verb-related speech high in %DET and high in RR. Child **(E)** produced high %DET but low RR. Child **(F)** produced low %DET but higher RR. Child **(G)** produced low %DET and low RR.

Each recurrence matrix was plotted to create a recurrence plot (RP; Marwan, 2008), which allows a qualitative inspection of how key features of sequential data change across time (see Figures 1, 2). Each point on the plot represents a single repeated item in the child’s production at different points across the transcript. In the present study, RP markings specifically indicate all points within a transcription in which the child repeats either a noun-related or verb-related lexical or grammatical item. For example, an RP for the lexical items in noun-related sequences with the text from Example 2 would pull out repetitions (represented as filled-in points) with the words *the*, *frogs*, and *city*.

If we had analyzed the lexical items in verb-related sequences, an RP for the same text would identify no recurrent sequences since no verb-related repetitions exist (e.g., exact repetitions of “*are gonna invade*” as a verb trigram). That is, there would be no recurring dots for these verb-related lexical items. However, RPs would pull out repetitions in the individual noun-related grammatical units (e.g., repeating determiners five times across Example 2) and verb-related grammatical units (e.g., repeating verbs seven times across Example 2). Thus, each diagonal line represents repetitions in sequences that the child produced at different times throughout the transcript. For instance, the noun phrase “the frogs” would be represented as a diagonal line on the RP since it is repeated twice verbatim.

#### Metrics

In addition to visual inspection, we can quantify the patterns and sequences of points on RPs to yield a variety of metrics. Here, we specifically focus on *recurrence rate* and *determinism*. Recurrence rate (RR) captures the percentage of the RP containing filled-in points (relative to all possible points); high RR indicates frequent reuse of lexical or grammatical units. For example, we could track “ice cream” in a single child’s transcript: “You got me *ice_cream*. Big ice_creams. You’ll have vanilla and I’ll have white *ice_cream*.” In this example, note that—since only *exact* repetitions would count as recurrent for noun-related lexical items—the word “ice cream” is only counted as repeating twice; the plural “ice creams” is not included. Low RR indicates infrequent reuse of the lexical or grammatical units (e.g., the word “big” in the previous example was only produced once).

When recurrent points occur in succession to create line structures, we can visualize a repeating trajectory. The percent of recurrent points on the RP that involve these diagonal line structures (i.e., two or more consecutive points) is known as percent determinism (also simply called determinism; DET). Determinism can reveal whether strings of repeated structures occur across the same contexts. Note that these repetitions themselves need not be sequential: That is, the repeated strings can occur across the entire transcript as well and are treated the same way. High %DET indicates that children frequently repeat the same lexical or grammatical combinations. For example, consider “ball” in this excerpt of a single child’s transcript:

#### Example 4

**Table:** 

Lexical:	Can I play with **the ball**?
Grammatical:	Modal-pronoun-verb-preposition-**determiner**-**noun**
Lexical:	Where’s **the ball**?
Grammatical:	**Pronoun**-**thirdpersonsingular**-**determiner**-**noun**
Lexical:	He likes balls.
Grammatical:	Pronoun-verb-thirdpersonsingular-**noun**-**plural**
Lexical:	Of dogs that like to play ball.
Grammatical:	**Preposition**-**noun**-**plural**-**pronoun**-verb-1-verb-noun
Lexical:	He likes to play with all **the balls**.
Grammatical:	Pronoun-verb-thirdpersonsingular-2-verb-**preposition**-**noun**-**determiner**-**noun**-**plural**
Lexical:	And mine too but I **do not let** him have **the balls** but I **do not let** him have **the balls** because.
Grammatical:	3-pronoun-adverb-4-pronoun-**auxiliary**-**verb**-pronoun-verb-**determiner**-**noun**-**plural**-5-pronoun-**auxiliary**-**verb**-pronoun-verb-**determiner**-**noun**-**plural**-6
Lexical:	There’s some balls that can that he can choke on it.
Grammatical:	**Pronoun**-**thirdpersonsingular**-noun-**noun**-**plural**-**pronoun**-pronoun-modal-verb-preposition-pronoun

Noun-related units referred to in the text explanation are in blue while verb-related units are in orange. The bolded darker blue (versus the non-bolded lighter blue) indicates that the noun-related lexical or grammatical units are a part of a deterministic sequence; the bolded darker orange (versus the non-bolded lighter orange) indicates that that verb-related lexical or grammatical units are a part of a deterministic sequence. Unlike in the prior examples, the black font in this example indicates that the words/grammatical units are not being counted as part of a deterministic structure. In this example, the child repeats “the ball” twice and “the balls” three times. A closer look at these phrases reveals that the child frequently combines grammatical units in the same way (e.g., preposition-noun; determiner-noun; determiner-noun-plural; noun-plural-pronoun). Lower determinism indicates that children are testing out many different unit combinations (e.g., only repeating the verb-related words “do not let” in this example).

The center line of each RP—the *line of identity* (LOI)—indicates lag-zero. By *lag-zero* (as it is called in autocorrelation), we mean all instances when that moment in the time series is compared to itself; this means that RR is always equal to 1 for the LOI. These self-comparison values do not vary across the children and are therefore ignored in RQA.

### Statistical analyses

All analyses were completed using R in RStudio (*version 1.1.423*; R Core Team, 2021). Current best practices for RQA were applied to the data (see Carello and Moreno, 2005; Riley and Van Orden, 2005). Our primary analytic approach was to use linear models to predict changes in RR and %DET, respectively, by Type (Noun-related vs. Verb-related) and Analysis Level (Grammar vs. Words). By also including more macro spontaneous speech metrics in the model (i.e., MLU and Total Number of Utterances,), we can account for variance directly from the structure of the children’s language, and we can directly compare the dynamical approach to the traditional approach. Autism Severity Score was included in the model to explore the degree to which repetitiveness was a facet of language development versus a characteristic of being autistic. Supplementary analyses controlling for NVIQ did not improve model fits when predicting either RR or %DET, and so NVIQ was not included in the models.

In interpreting RQA results, it is important to note that many metrics are not inherently meaningful. That is, they are often more useful as relative metrics compared across conditions (e.g., between experimental conditions, between two interlocutors) *via* inferential statistics. However, this could be potentially problematic in the case of understanding whether the observed values differ from those values that might be expected simply by chance. We address this concern using *approximate permutation tests*, which allow a researcher to create and test surrogate time series (i.e., use itself as a baseline; see Chiovaro et al., 2021, and Paxton and Dale, 2017). Permutation tests go beyond the raw frequencies of categories to test the degree to which the *structure* of the categories across the transcript can be found together more often than would be expected by chance (i.e., the baseline). Through these permutation tests, we can evaluate whether categories of words and grammatical units are organized in meaningful ways.

Here, we conducted tests for significance with confidence intervals at the upper and lower bounds of the 95th percentile (comparable to alpha criteria of 0.05). We then created 100 permutations of each participant’s transcript (i.e., removing category dependencies across the transcript but maintaining raw frequencies) and conducted RQA on each of these permutations. We compared this output to what we might expect to see by chance, again preserving the participant-level variability (i.e., comparing the observed values from a given participant to the permutation values created from that same participant’s data). The proportion of times that the real-time series’ values exceed the baseline time series’ values is used as the alpha criterion for significance. However, because we maintain the frequencies of the original time series, it is critical to note that permutation tests can *only* be used to establish baselines for RQA metrics that rely on sequences—here, meaning that we can only examine %DET and not RR. Of the permutation tests run for the %DET of noun-related and verb-related words and grammar, respectively (i.e., four measures), we find that 80.39% of noun-related grammatical unit data (*n* = 51; *p_median_* < 0.001, *p_sd_* = 0.22), 89.36% of the noun-related lexical data (*n* = 47; *p_median_* < 0.001; *p_sd_* = 0.13), 90% of the verb-related grammatical data (*n* = 50; *p_median_* < 0.001; *p_sd_* = 0.04), and 97.92% of the verb-related lexical data (*n* = 48; *p_median_* < 0.001; *p_sd_* = 0.02) are above the criterion. This means that, in general, the observed structures within the data tend to appear together more than what would be expected by chance.

## Results

### Characterizing the sequences of grammatical units

Since there are so many possible lexical units within any given noun- or verb-related construction and many fewer possible grammatical units, we only show visualizations of the grammatical unit data. The left-hand side of Figures 1, 2 show sequence figures to characterize children’s production of syntactic and morphological units across the span of a single transcript. Note that since each child may vary in the number of grammatical units that they might produce, their x-axes can vary. Each point represents a single syntactic or morphological unit and the order in which they occur (and reoccur) over the course of a transcript. Each sequence figure shows the sheer quantity of units that a single child produces.

Figures 1, 2 also highlight differences in the degree to which children use certain grammatical items within the same ADOS protocol. For instance, children A and D from Figure 1 produce speech that is similar in quantity (i.e., number of utterances and number of noun-related grammatical units); however, D produced a wider range of grammatical units overall (see full sequence plots). For instance, D produced many more pronouns, determiners, wh-questions, and prepositions overall, while A produced many more nouns and number units. In contrast, B produced fewer utterances than A and D but still produced a wide range of grammatical units. Child C produced the fewest utterances and the fewest grammatical units (i.e., did not produce proper nouns, adjectives, gerunds, numbers, prepositions, or plurals).

Figure 2’s full sequence plots show that even children who are more similar in utterance quantity (i.e., children F and G) may produce similar numbers of verb-related grammatical units. For instance, while child F produced prepositions, the negative, the 1st- or 3rd-person singular, the past participle, the past tense, and the auxiliary, child G did not. Thus, child F produced more instances and a greater variety of verb-related grammatical units. Children E and G highlight the opposite pattern: Child E produced far fewer utterances than both F and G; however, although E and G produced a different number of utterances, they both similarly produced a small number of grammatical units, especially relative to F. Sequence graphs thus show that RQA is a good measure to capture the differences in how children produce their noun-related and verb-related sequences.

To the right of the sequence plots in Figures 1, 2 are the example corresponding recurrence plots (RPs). RPs also highlight the variability of units within our sample. Again, note that the *x*- and *y*-axes represent the categories (word or grammatical units) that children produce across the sequence of a transcript. Because each child may vary in the number of different grammatical units that they produce, their *x*- and *y*-axes differ on the RPs; accordingly, this means that the size of a single point will be larger or smaller on the graph, depending on the total number of possible points. A filled-in space indicates that the child is revisiting a previously used category (i.e., contributing to RR), while the line structures indicate that the child is revisiting a previously used sequence of categories (i.e., contributing to %DET).

The RPs in Figure 1 show that Child A repeats many unit combinations verbatim (possibly indicating less advanced production), whereas B repeats less but often repeats combinations of “preposition determiner noun” and “determiner noun plural” (indicating more advanced production; see sequence plot of first 50 units). C repeats words often but produces few new words across many new sequences (indicating moderately advanced production; e.g., “determiner noun” and “noun”), whereas D keeps using new units in new combinations without revisiting prior ones (indicating moderately advanced production; e.g., “determiner noun” versus “preposition pronoun”). This contrast between C and D is particularly striking in the sequence plots for their first 50 grammatical units. That is, these plots show that C repeats a few units (i.e., common noun and pronoun) quite frequently; D only repeats pronouns frequently. The RPs for verb-related words and grammar were calculated in the same manner.

### Recurrence rate (RR)

**Figure 3:**
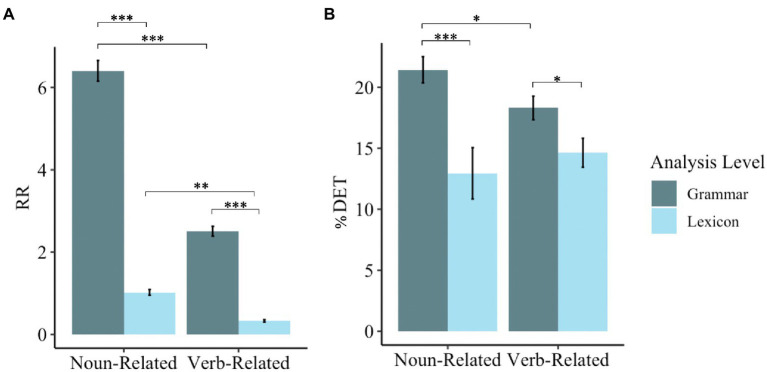
RQA metrics for the components of noun-related and verb-related phrases. *** = *p* < 0.001; ** = *p* < 0.01; * = *p* < 0.05. Error bars represent standard errors. **(A)** Shows the mean recurrence rate across the sample. **(B)** Shows the mean percent determinism across the sample.

Our analyses examined the degree to which children tend to reuse the same lexical or grammatical units (i.e., RR) by Type (Noun-related vs. Verb-related) and whether these RR values correlated with the Total Number of Utterances, MLU, and ADOS Severity Score at that visit. Descriptive data for RR by Type (Noun-related vs. Verb-related) and Analysis Level (Lexicon vs. Grammar) are provided in Table 5. These data are visualized in Figure 3. Note that the predictor variable Total Number of Utterances was moderately correlated with both MLU and ADOS Severity Score, whereas MLU and ADOS Severity Score were strongly correlated with one another (see Table 6).

**Table 5:** Means and standard deviations for RQA metrics.

Phrase type	Measure	M (SD)	Lower 95% CI	Upper 95% CI
Noun	Lexicon RR	1.02 (0.49)	0.74	1.30
	Grammar RR	6.40 (1.78)	6.12	6.68
	Lexicon %DET	12.94 (15.05)	10.20	15.70
	Grammar %DET	21.44 (7.67)	18.70	24.20
Verb	Lexicon RR	0.33 (0.20)	0.05	0.61
	Grammar RR	2.51 (0.86)	2.23	2.79
	Lexicon %DET	14.63 (8.44)	11.90	17.40
	Grammar %DET	18.31 (6.87)	15.5	21.10

**Table 6:** Correlations between predictor variables.

Measure	1	2	3	4
1. Type				
2. Level of analysis	0			
3. MLU	0	0		
4. Total utterances	0	0	0.32***	
5. ADOS severity score	0	0	−0.59***	−0.21***

Each value represents a correlation coefficient (*r*). ***indicates *p* < 0.001.

Linear modeling was carried out to investigate whether these variables could significantly predict RR.^.^ Results indicated that the model explained 87.32% of the variance in RR and that the model was a significant predictor of RR, *F*(15,188) = 94.17, *p* < 0.001. For clarity and flow, model results—including unstandardized betas and confidence intervals—can be found in Table 7 rather than in the text.

**Table 7:** Regression results for the model predicting RR.

Predictor	B	lower 95% CI	upper 95% CI	SE	*t*
(Intercept)	6.93***	5.22	8.62	0.87	7.97
Type	−3.88**	−6.31	−1.45	1.23	−3.16
Analysis level	−6.23***	−8.66	−3.81	1.23	−5.07
MLU	−0.40***	−0.60	−0.20	0.10	−3.91
Total utterances	0	−0.01	0	0.002	−0.47
ADOS severity score	0.20**	0.05	0.35	0.08	2.63
Type: Analysis level	3.55*	0.12	6.98	1.74	2.04
Type: MLU	0.35*	0.06	0.63	0.14	2.14
Type: Total utterances	0	−0.01	0	0.003	−0.69
Type: ADOS severity score	−0.18	−0.39	0.03	0.11	−1.68
Analysis level: MLU	0.40**	0.11	0.68	0.14	2.74
Analysis level: Total utterances	0	−0.01	0.01	0.003	0.11
Analysis level: ADOS severity score	−0.13	−0.35	0.08	0.11	−1.25
Type: Analysis level: MLU	−0.35	−0.75	0.06	0.01	−1.69
Type: Analysis level: Total utterances	0	−0.01	0.01	0.005	0.35
Type: Analysis level: ADOS severity score	0.14	−0.16	0.44	0.15	0.91

*R*^2^ = 0.873***. *indicates *p* < 0.05. **indicates *p* < 0.01. ***indicates *p* < 0.001.

The analysis revealed a main effect of Type, such that noun-related speech involved a higher RR (*M* = 3.71, *SD* = 3.00) than verb-related speech (*M* = 1.42, *SD* = 1.26). We found a main effect of Analysis Level, in which the RR of grammatical units (*M* = 4.46, *SD* = 2.40) was higher than the RR of lexical units (*M* = 0.68, *SD* = 0.51). The two-way interaction between Type and Analysis Level was not significant; however, based on visual inspection of Figure 3, Panel A, we conducted follow-up analyses Tukey’s *post-hoc t*-tests comparing the RR of noun-related and verb-related grammatical and lexical units. Our analyses revealed that RR was significantly higher for noun-related words than verb-related words [*B* = 0.70, *t*(176) = 3.91, *p* < 0.01] and for noun-related grammar than verb-related grammar [*B* = 3.89, *t*(176) = 21.81, *p* < 0.001]. Results also revealed a higher RR for noun-related grammar than noun-related words, *B* = 5.38, *t*(176) = 30.15, *p* < 0.001. Similarly, RR was higher for verb-related grammar than verb-related words, *B* = 2.19, *t*(176) = 12.24, *p* < 0.001.

**Figure 4:**
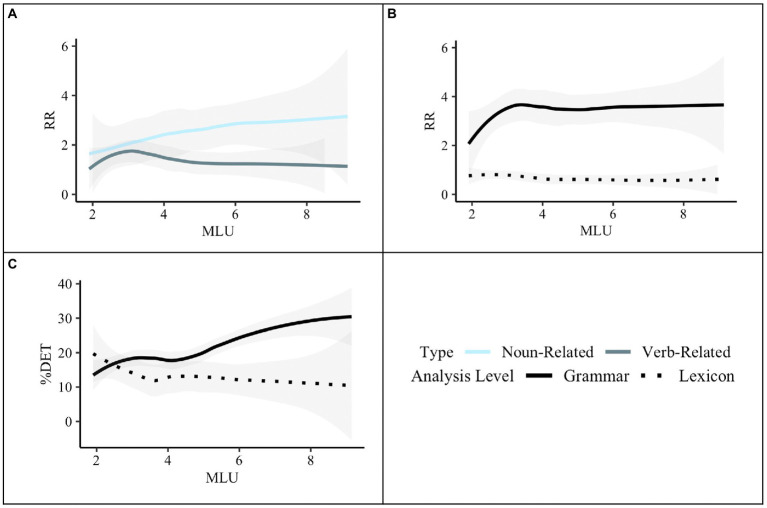
Predictors for RR and %DET from mixed effects modeling. Panel **(A)**, shows predictors for RR by type and MLU. Panel **(B)**, shows predictors for RR by the level of analysis and MLU. Panel **(C)**, shows predictors for %DET by Level-of-Analysis and MLU. Panels **(B)** and **(C)**, show the RR and %DET, respectively, for grammar and lexicon collapsing across noun-related and verb-related items, whereas Panel **(A)**, shows the RR for the noun-related and verb-related items collapsing across grammar and lexicon.

Generally speaking, the analysis further revealed that the Total Number of Utterances was not related to RR in any way. More specifically, we found a main effect of MLU, with MLU increasing as RR decreases. Results revealed a Type-by-MLU interaction. Interactions are visualized in Figure 4. While RR does not vary for verb-related items by MLU, it does for noun-related items, with RR lower for noun-related items when MLU is short (see Figure 4, Panel A). Furthermore, we found a significant Analysis-Level-by-MLU interaction: Although RR and MLU do not change according to lexical units, RR of grammatical units is positively correlated with MLU when the MLU is short but plateaus when MLU is longer (see Figure 4, Panel B). Results also revealed that ADOS Severity Scores positively predicted overall RR.

### Percent determinism (%DET)

**Figure 5:**
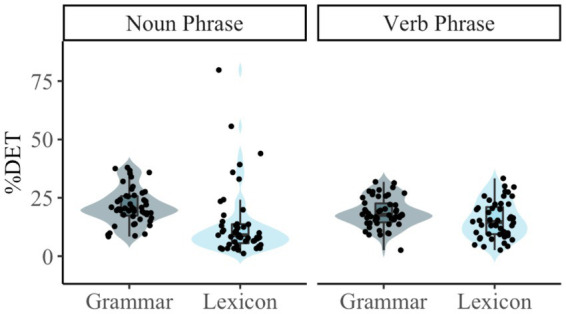
Variability in children’s combinations of phrasal units by analysis level. One child is combining the same noun-related words over and over (e.g., “Happy Birthday,” “Scooby Doo snack,” “upside down”). However, this child only produced 35 utterances, most of which were very short (MLU of 2.67) and so, although they produced the highest word determinism, even adding one new grammatical unit to their noun-related lexical repetitions would make them less grammatically deterministic (e.g., the child said “pronoun-adverb-adverb and noun-adverb-adverb”). This same child also produced the highest percent of echolalic utterances in the sample. Similarly, five other children repeated the same words in noun phrases. Most of the other children generated a lot of possible word combinations for noun phrases in each utterance.

We examined characteristics of the degree to which units tend to fall on repeated sequences of the same grouping of units (i.e., *%*DET) by Type, Analysis Level, MLU, Total Number of Utterances, and ADOS Severity Score. The lexicon *%*DET values capture how much the children are combining the same words that are in noun-related and verb-related sequences, respectively, in the same way (i.e., productivity). Figure 5 shows the variability in the *%*DET of the 51 children.

Visual inspection of the figure reveals that children seem to be more productive in their verb-related lexicon than in their noun-related lexicon, as demonstrated by the fewer children repeating the same lexical sequences in their verb-related constructions. Example 4 (above) shows an excerpt from one child and highlights this difference in the *%*DET of noun-related and verb-related lexicons. In blue bold ink are the repeating noun-related word combinations and grammar combinations. In contrast, the orange bold ink highlights the repeating verb-related word combinations and grammar combinations. While some children reuse word combinations frequently (e.g., “the balls” in Example 4; see Figure 4), in general, it is to a much smaller extent than the degree to which they repeat grammatical combinations (e.g., determiner-noun-plural and auxiliary-verb). Note that while several noun-related combinations are repeated in Example 4, many combinations are new. Descriptive data for *%*DET by Type and Analysis Level are provided in Table 5.

We used linear regression to test whether main effects of and interactions between Type, Analysis Level, MLU, Total Number of Utterances, and ADOS Severity Score predicted variance in *%*DET. The model was statistically significant, *F*(15,188) = 3.40, *p* < 0.001, *R*^2^ = 0.15. As with our analyses of RR, we present all model results—including unstandardized betas and confidence intervals corresponding to main effects and interactions—in Table 8.

**Table 8:** Regression results predicting %DET.

Predictor	B	Lower 95% CI	Upper 95% CI	SE	*t*
(Intercept)	1.80	−16.36	19.97	9.21	0.20
Type	3.49	−22.20	29.18	13.02	0.27
Analysis level	6.57	−19.12	32.26	13.02	0.50
MLU	3.10**	0.97	5.24	1.08	2.87
Total utterances	−0.03	−0.08	0.02	0.03	−1.10
ADOS severity score	1.48^✝^	−0.11	3.07	0.81	1.84
Type: Analysis level	9.73	−26.60	46.06	18.42	0.53
Type: MLU	0.12	−2.90	3.14	1.53	0.08
Type: Total utterances	0.01	−0.07	0.08	0.04	0.18
Type: ADOS severity score	−1.19	−3.44	1.05	1.14	−1.05
Analysis level: MLU	−2.89^✝^	−5.91	0.13	1.53	−1.89
Analysis level: Total utterances	−0.01	−0.08	0.07	0.04	−0.15
Analysis level: ADOS severity score	−0.29	−2.53	1.96	1.14	−0.25
Type: Analysis level: MLU	−1.50	−5.77	2.77	2.16	−0.70
Type: Analysis level: Total utterances	0.02	−0.08	0.13	0.05	0.43
Type: Analysis level: ADOS severity score	−0.19	−3.37	2.99	1.61	−0.12

*R*^2^ = 0.150***. ^✝^indicated *p* < 0.07. *indicates *p* < 0.05. ***indicates *p* < 0.001.

Type did not significantly predict *%*DET (*p* = 0.79), suggesting that children did not vary in the degree to which they combined units for noun-related and verb-related words and grammar. Children also did not alter their deterministic productions by analysis level (*p* = 0.61). However, based on visual inspection of Figure 3B, we conducted follow-up *t*-tests comparing %DET for words and grammar of noun-related and verb-related units. Results revealed a higher %DET for grammatical units compared to lexical units for both noun-related units [*t*(50) = 3.63, *p* < 0.001] and verb-related units [*t*(50) = 2.47, *p* < 0.05]. We also found a higher %DET for noun-related grammar than verb-related grammar (*p* < 0.05, *d* = 0.36), but this did not hold for words (*p* = 0.38). In general, MLU was positively associated with *%*DET. This association only emerged once MLU reached approximately 4. Moreover, we found a trending interaction between Level of Analysis and MLU (*p* = 0.06) (see Figure. 4, Panel C). Although the %DET of lexical units does not vary by MLU, it does for grammatical units, with %DET higher for grammatical units when MLU is larger. Finally, we also found a trending positive association between %DET and ADOS Severity Score (*p* = 0.067). No other main effects or interactions were statistically significant.

## Discussion

The current study presented an innovative technique (i.e., recurrence quantification analysis) for measuring the productivity of syntax; this technique can consider the dynamic nature of syntax and the variability in how productivity unfolds in running conversations. RQA provided a way to capture gradations of repetitions (e.g., quantity, diversity, and sequences) to shed light on a wide spectrum of language use in children with ASD. For instance, using this technique, we explored individual differences in the productivity of noun-related and verb-related speech. Our first hypothesis was not supported since we found that degree of talk and recurrence metrics were unrelated.

In contrast, our findings were consistent with our second hypothesis, that recurrence measures would be associated with MLU. Our major finding here was that RR was related to MLU, as children with higher MLUs repeated noun-related grammatical units *less* across the entire MLU range, while children who repeated verb-related grammatical units *more* produced longer utterances but only up to MLUs of 3–4. Notably, determinism provided an even more detailed look into the structures that make up productivity than are made possible by traditional composite linguistic measures. For example, while determinism was not related to total number of utterances, it was related to MLU, thus lending even more credibility to our second hypothesis. This finding highlights how challenging it is to establish productivity in children who consistently produce short utterances. Children with ASD who produced longer utterances manifested more productivity; thus, they were not just repeating the same utterances over and over. Furthermore, the determinism of grammatical units was what seemed to drive this relationship with MLU. That is, children who repeated grammatical combinations also produced more complex language, signifying the importance of creating varied grammatical constructions for early productivity.

We also investigated how RQA measures compared to well-established linguistic analyses in a sample of 5-year-olds with autism from the Autism Phenome Project dataset (Wittke et al., 2017). Our analyses revealed that the recurrence rate of grammatical and lexical units within noun-related and verb-related speech mapped onto traditional linguistic analyses; for example, grammatical units were repeated more than lexical units. Measures of determinism further illuminated gradations in the productivity of grammatical language use for children with ASD. As expected, grammar was more productive (i.e., higher %DET) than words in both noun-related and verb-related speech sequences. Noun-related grammar usage was more productive than verb-related grammar usage, but no significant noun-verb differences were found for words. Thus, RQA and traditional linguistic analyses—at least to some extent—identify similar signals.

In broad brush, our findings are consistent with the elicited bootstrapping hypothesis. Although we did not directly measure social motivation nor attempted to test the complete theoretical model, we consider autism severity scores a parallel to social motivation (see also Naigles and Chin, 2015; Thomas et al., 2022), and our results showed that children with less social motivation were more repetitive. That is, they repeated sequences of words more as well as individual words more, either immediately or further along in the conversation. In what follows, we explore possible explanations for why specific patterns of repetition emerged across the different types of speech and levels of analysis, and consider possible explanations for the reported associations between recurrence measures and traditional linguistic measures.

### Recurrence metrics relate to productivity

#### Recurrence does not equate to being more (or less) talkative

In general, we found no association between the number of utterances and either recurrence metrics (i.e., RR and %DET). This is plausible given that producing fewer utterances does not mean that the children are not producing rich utterances when they do talk. For instance, two children in our sample produced only 29 utterances but varied in the complexity of those utterances. One child repeated noun-related grammatical units moderately (e.g., “Ah, I do not pop *bubbles*. *Bubbles* go. Ah, *bubbles* pop pop,”), while the other repeated noun-related grammatical units frequently but had more complex language (e.g., “*I* want to play balloon. *I* want *the mommy*’s phone. Clean up *the toys*”).

#### Recurrence captures individual differences in productivity

We did find that children who repeated grammatical units more frequently (e.g., more Determiner-Noun or Verb-ing sequences) produced *longer* utterances overall. In a way, this is necessary, as the repetition of grammatical units means that there are indeed sequences of units, hence longer utterances. This was particularly evident when the children’s speech was in the early phases of becoming more complex.

However, it seems that RR only matters for the onset of grammatical speech and then the relationship plateaus, with a lot of variation in repetitions for high MLU (see Figure 4B). Possibly there is a plateau because RR does not differentiate between the child who just says Determiner-Noun all the time versus the child who says Determiner-Noun and Verb-ing, which would be captured by %DET. Thus, this shift in patterning likely reflects the shift over to multiword speech. There are a few reasons this might occur. First, it could be that there are just fewer children with larger MLUs driving this effect. However, the distribution of scores in grammatical unit repetition (see Figure 4) indicates that this is not the case.

Second, perhaps at first children produce lots of pronouns, which keeps their MLU short. For instance, a child who produces less complex speech might be more likely to frequently say “get *it.*” For these children, relying on these specific grammatical units may hamper or delay their production of longer utterances. Thus, children’s longer utterances do not just involve saying the same items over and over. Rather, longer utterances involve—in ASD as in TD-—fairly morphologically or syntactically rich sentences (e.g., sentences with multiple clauses that contain adjectives, prepositions, adverbs, and verbs).

Finally, it could be that since there are only so many repetitions in noun-related speech that one can do in the span of English grammar. After a certain point, there is only a set number of ways that English can support noun-related grammatical recurrence. For instance, one could produce sentences with determiner-noun or determiner-adjective-noun to form a legal noun phrase; however, adjective-determiner-preposition-noun would not be an appropriate noun phrase construction in the English language. So, if the child is repeating lots of noun-related grammatical units then they are probably producing shorter utterances and if they are producing shorter utterances then they are probably repeating a lot of noun-related grammatical units. Repeating noun-related grammatical units (e.g., “A baby. A firetruck. A boy. With the pants.”) does not enable the child to produce longer utterances, because it is the verbs that extend the utterance length (e.g., “They will not stand up. Yeah they were eating. And then they come in. And they took the food away.”). Either way, the data suggest that these children may benefit from therapy to address verb-related speech.

Perhaps the closest analog to our own study is Lieven et al. (2009), who focused on the productions of four TD children. Consistent with our findings, they reported that noun-related (so-called REF) repetitions are more frequent than verb-related (so-called PROCESS) repetitions. In contrast to the current findings, they also report generally less repetitiveness (i.e., fewer repeated multi-word utterances) in children with higher MLUs (and across development for one child, with their MLU changing from 1.6 to 2.2). Slight differences between our results and Lieven et al.’s (2009) research may be due to their decision to confine analyses to multi-word utterances that have been repeated (which means they did not count repetitions of single words that might appear across utterances), their use of a traceback method (which means they had to more arbitrarily decide what was versus was not repeated), and their four-child sample size (which resulted in a much smaller MLU range of 1.6–2.2). Upon analyzing the data further by type (i.e., noun-related speech versus verb-related speech) they revealed that PROCESS-related/verb-related multi-word repetitions increase with MLU, which potentially matches our increase from an MLU of 2–4. It could be that this association between verb-related grammatical units and MLU reflects a shift from learning how to combine basic words to learning how to combine grammar in more complex ways. This would indicate that the value of RR may lie in its potential to capture emerging complexity in grammatical constructions, but beyond this shift, RR is less informative.

This interpretation of the data is partially consistent with the hypothesis of predictive impairment in ASD (Sinha et al., 2014); one component of this hypothesis suggests that challenges in prediction lead to overly repetitive behavior as compensation. Similarly, our analyses found that higher RRs for noun-related structures were associated with more advanced language; however, high RRs for verb-related structures did not yield the same expected association. Note, though, that all children repeated themselves at least somewhat; we conjecture that it was the children who found *a few structures* to consistently refer back to while testing new structures who were the ones with better language abilities. Therefore, all of the differences in methods considered, the data broadly suggest that our children with ASD are not markedly different from the TD children in their RR; variations in findings are likely based on the language level (MLU), not ASD presence (see also Weismer and Saffran 2022).

Interestingly, the only significant relationship that emerged with %DET was MLU, as children producing longer utterances combined the same grammatical units more frequently, showing more advanced productivity since they are practicing the same sentence structures. For example, a child with an MLU of 3.04 repeated the sequence “started to took off” frequently while saying “Then it started to take off. The. To took off. It started to took off already.” Notice that this child is building on each of the repeated sequences in different ways across each sentence. In contrast, higher MLU children combined the same grammatical units more frequently (i.e., showing more advanced productivity). For instance, the child from Example 4 had an MLU of 5.29 and repeated determiner-noun-plural sequences several times within the brief excerpt. Our results suggest that determinism goes beyond frequency counts, providing more detail on the structure of productivity. Not only do children with ASD vary in their usage of words and grammatical units, but—depending on their language skills—children with ASD exhibit different dynamics in their speech patterns, too.

### Recurrence measures mirror and extend traditional linguistic analyses

Across the children in this study, a large degree of variability was evident in the repetitions of words and grammatical units in noun-related and verb-related speech, in amount of talk, in types of words and grammatical units, and in combinations of these units. This variability is consistent with previous work documenting a vast heterogeneity in the language skills of children with ASD and this variability spans their lexicon, syntax, and morphology (Kjelgaard and Tager-Flusberg, 2001; Modyanova et al., 2017). This variability is perhaps not surprising given existing work claiming that motivations to communicate may actually alter the degree to which children on the autism spectrum exhibit repetitive speech (i.e., elicited production theory; Camarata and Yoder, 2002; Sameroff, 2009). Thus, in our language sample, we may possibly be capturing these differences in motivation to communicate across the different activities. However, these data cannot parse out whether repetitions occur because the child is problem-solving their social partner’s intent, affirming their preference by imitating or producing a self-regulatory behavior (i.e., stimming).

#### Increased recurrence of nouns and grammatical structures

We found more repetitions in noun-related speech than verb-related speech, of noun-related words than verb-related words, and of grammatical units than lexical units. Such findings are consistent with the structure of the English language, of our choice of lexical and grammatical units, and of the ADOS protocol. A closer look at type and token distributions of units in noun-related and verb-related speech can help explain why these patterns might emerge.

The data revealed that differences in repetitions by speech type may emerge because children tended to produce many different noun-related words (average number of noun-related word tokens = 267, range = 27–636 words), but only a few of these units were repeated frequently. By contrast, children produced fewer verb-related words overall (average number of verb-related word tokens = 145, range = 12–338 words) but repeated a greater variety of them. These differences in variety and volubility in noun- and verb-related production are consistent with other research on TD children. For instance, researchers have found that of the earliest words that TD children produce, over half are nouns, while less than 25% are verbs (Stern, 1924; Nelson, 1973; Fenson et al., 1994). Further, TD children produce many more noun types (see Sandhofer et al., 2000) and more noun tokens (Tardif et al., 1997).

Another possible explanation for these findings is that the noun-related units are largely syntactic (10 possible syntactic items versus 4 possible morphological items), whereas the verb-related units are mostly morphological, not fully syntactic (5 possible syntactic items versus 11 possible morphological items). That is, fewer grammatical items comprised noun-related speech (i.e., 14 possible items) than verb-related speech (i.e., 16 possible items; see Tables 3, 4), leading to more repetitions in noun-related speech. Finally, because there are fewer grammatical items than lexical items (in both noun and verb phrases), it is unsurprising that RR is lower for lexicon than grammar (see Naigles et al., 2009, for documentation of productivity in verbs). These analyses, therefore, show that RR is capable of capturing the difference between noun-related and verb-related speech and grammar and lexicon and so analyses are consistent with traditional linguistic analyses.

Our finding that children more frequently combined grammatical units in the same ways compared to word units, for both noun-related and verb-related speech, likely emerged because there are simply many more words that children could choose to combine compared to grammatical units (i.e., “a cute dog” would be flagged as a different combination than “a fluffy dog”). We also found that children combined noun-related grammatical units more so than verb-related grammatical units, but this difference in speech type did not hold for words. This is likely a facet of our coding, in that we coded for more ways to appropriately combine grammatical units of noun-related speech than verb-related speech, given our choice to not code for verb argument structure.

### Recurrence captures autism symptomatology

Our analyses exploring the relationships between RR, %DET, and autism diagnosis-related metrics revealed some interesting nuances to help explain extant research. Primarily, we found that children who were generally more repetitive tended to present with more autism traits; this matches the broader ASD literature, which suggests that repetitive behaviors are common in ASD (Tager-Flusberg and Calkins, 1990; American Psychiatric Association, 2013). Thus, calculating and comparing RRs of speech for both autistic and non-autistic individuals could further help refine the prediction impairment hypothesis (Weismer and Saffran, 2022).

We also build on the existing literature about language profiles in 4- to 8-year-old children with autism (see Van Santen et al., 2013; Thomas et al., 2022). For instance, Van Santen et al. (2013) reported no difference in intra-turn self-repeats of words by autism diagnosis. However, we found that children who were generally more repetitive (across lexical and grammatical units) tended to demonstrate more autistic traits, suggesting that Van Santen et al. (2013) may have not captured the relevant metric of repetition. Thus, we add that like repetitions at the lexical level, repetitions at a finer granularity of measurement (i.e., grammatical units and different parts of speech) may also provide informative data points to understand differences across the spectrum. We extend previous findings by including ASD participants with a wider range of IQ scores and participants ranging from talkative to minimally talkative (whereas Van Santen et al. exclusively focused on low verbal children), making the current findings more representative of the ASD population.

### Limitations and future directions

While these results are intriguing, there are several limitations within the present study. First, the current data did not include any comparison groups for the ASD group, making it difficult to assess the degree to which variability in recurrence is unique to autism or characteristic of broader language heterogeneity in all children. To better describe the productivity of syntax in autism, it would be important to conduct studies that involved a TD group, a Developmental Language Disorder group, more age groups, a language-matched group, and an age-matched group.

Second, these data are drawn just from interactions during the ADOS, with the child engaging with a clinician. However, child speech and more importantly, the degree to which that speech is repetitive, can vary by interactional context. For instance, Gladfelter and VanZuiden (2020) found that school-aged children with ASD repeated themselves less frequently (i.e., self-repeating) during storytelling compared to during play-based contexts. These findings suggest that context can shape the degree to which children repeat: more unstructured contexts, as in the current study, may involve more lexical repetitions, which could be an indicator of less productive speech (see also Kover et al., 2014, for differences in the number of unique words by context). This raises the possibility that the language samples collected in the ADOS can underestimate linguistic complexity. Relatedly, Naigles et al. (2009) found more productivity in the verb use of TD children within parent diaries, presumably because this format required all verb use to be written down across the children’s daily activities. Work across a variety of contexts, therefore, suggests a broader need to study language in autism within more naturalistic and a wider variety of settings. Perhaps this issue could be tackled *via* the LENA system, which can capture many settings of talk at home. At present, LENA recordings are not as well analyzed as traditional free-play interactions, as LENA outputs the presence of speech and auto-generates word counts but not types of words, syntactic complexity, or transcriptions of the speech itself. Furthermore, research has suggested that it is not yet useful for detecting speech vocalizations in ASD (Wang et al., 2017; Jones et al., 2019; Sulek et al., 2022). Although LENA’s raw data are not yet amenable to lexical or grammatical RQA, LENA transcripts could reveal what the child is saying over the entire day. Coding these transcripts for RQA would be an important next step in this avenue of research.

Third, given our focus on child language production, we did not assess the role of the social partner in prompting repetitive or productive speech. However, across our sample, there was large variability in the degree to which parents were present and involved for ADOS administrations. The degree to which this social partner, and even the clinicians and the experimenters, contribute to the reported patterns is unclear. Further characterization of recurrence in speech should involve more conversations with parents (see Fusaroli et al., 2020, Unpublished manuscript), cross-recurrence with different conversational partners (e.g., parents, clinicians, and strangers), and a comparison to intra-child recurrence for TD groups (see Dale and Spivey, 2006; Müller-Frommeyer et al., 2019). This type of work could be applied to analyze coherence in content within speakers (e.g., auto-scoring essays; Angus et al., 2012). It could also be helpful for assessing the degree to which speakers are on the same page (i.e., semantic alignment; Dale and Spivey, 2006; Fusaroli et al., 2020, Unpublished manuscript). Relatedly, we also have not considered how self-repetition, studied here, relates to echolalia, or the repetition of the speech of others. In our sample, only a few children produced a substantial number of echolalic utterances, and their RRs varied hugely, so drawing conclusions about this relationship was unwarranted. However, with a bigger sample of children producing more echolalic utterances, the relationship with self-repetitions could be studied in more depth.

Fourth, we have not included analyses that might map RQA metrics onto the subgroups that Wittke et al. (2017) first identified. Since the proportion of ungrammatical utterances (which was a key grouping variable for Wittke et al., 2017) correlated with RQA metrics, we might expect that RQA could pull out additional things from the subgroups to characterize these children in even greater detail.

Finally, our particular interests in understanding repetitions in noun-related and verb-related speech led us to remove all other data from consideration in our analyses and consider only two kinds of RQA metrics. This coding made it impossible to tell whether other parts of speech generate unique recurrence patterns. However, clearly, there are many other parts of speech (e.g., prepositional phrases and adverbial phrases). Other researchers have started to look at recurrence in grammar (Dale and Spivey, 2006; Müller-Frommeyer et al., 2019) but have not yet assessed all parts of speech. One approach to examining categories of speech might be to use recurrence block representation analyses created by Xu and Yu (2016). This approach would generate recurrence plots that showed where in time certain categories were chunked. Future work should also examine other RQA metrics not analyzed here; for example, in taking a dynamical systems approach, it may be valuable to explore attractor strength (or the relative “pull” of different kinds of behaviors) through the RQA metric known as maximum line length (or maxline; e.g., Pellecchia et al., 2005). While outside of the scope of the current article, future exploratory or confirmatory analyses of RQA metrics may provide valuable insights into these and similar phenomena.

## Conclusion

Autistic individuals comprise a diverse population with a diverse set of skills. This study is a first step in understanding the real-time syntactic structures that characterize the diverse range of language abilities in young children with ASD. While the current study did not attempt to model the entire elicited bootstrapping theory framework, we affirm that differences in early social motivation prompt a series of shifts in children with ASD’s language production and reciprocal language input. Based on the recurring patterns of grammar and lexicon observed within a rich, naturalistic, spontaneous language sampling opportunity, we emphasize that these productions were still characterized by complex and adaptive content not restricted to repetitive speech or echolalia. Results suggest that we should perhaps refocus from aggregate measures to consider many of the nuanced patterns that emerge across the span of a conversation.

The primary contribution of the current study is a technique for quantifying patterns of repetition in language automatically. This type of technique could help guide assessments and interventions in capturing and tapping into underlying mechanisms of repetitive language use in autism. That is, findings from this work, if replicated, may assist clinicians design more powerfully targeted therapies for developing early language use. Our RQA analyses showed that both grammatical productivity and lexical productivity were related to language competence in different ways to this heterogeneous sample of children with ASD. Beyond more traditional measures like MLU, it appears that *less* repetition in noun-related grammar leads to longer utterances, whereas *more* repetition of verb-related grammar leads to longer utterances (up to MLUs of 3–4 morphemes). This could benefit clinicians to more strategically structure their language interventions, working on increasing the diversity of lexical items while emphasizing the importance of grammatical repetition, particularly for verb-related units. A parallel in this treatment philosophy is seen in harnessing statistical learning for children with specific language impairment (SLI), now more commonly known as developmental language disorder (DLD; see Plante et al., 2014 and Plante and Gomez, 2018 for more information). We also suggest that—although it is important to capture simple single unit repetitions (i.e., repetitive speech and RR)—measures of how children combine these units (i.e., %DET) can shed light on how children are building their sentences (i.e., testing out new structures versus relying on the same structures over and over again). Findings ultimately suggest that fine-grained measures such as RQA metrics may have the power to illuminate this continuum of productivity in children with ASD.

## Data availability statement

Datasets on which analyses were performed are available at https://github.com/amandamankovich/ASD-Recurrence-Analysis. The raw data supporting the conclusions of this article will be made available by the authors, after consultation for privacy reasons with the APP team at the MIND Institute.

## Author contributions

AMM was part of the team that designed the original data collection. AM, KW, AP, and LN worked together on the design, coding, and analyses of the current study. AP developed the RQA code in R and RStudio. AM, JB, and LN worked together on the primary write-up of this study. All authors contributed to the article and approved the submitted version.

## Funding

This research was funded by NSF-IGERT DGE-1144399 to the University of Connecticut in addition to NIH R01 MH089626-1 and NIMH to the MIND Institute, Autism Phenome Project. AM was awarded a UConn Science of Learning Training Fellowship.

## Conflict of interest

The authors declare that the research was conducted in the absence of any commercial or financial relationships that could be construed as a potential conflict of interest.

## Publisher’s note

All claims expressed in this article are solely those of the authors and do not necessarily represent those of their affiliated organizations, or those of the publisher, the editors and the reviewers. Any product that may be evaluated in this article, or claim that may be made by its manufacturer, is not guaranteed or endorsed by the publisher.

## Footnotes

^1^It is straightforward to assess productivity within comprehension/novel word studies, such as those that investigate whether children can use sentence structures to figure out the meaning of unknown words. Indeed, children can use sentence structures to identify words (e.g., for TD research, see Naigles et al., 2005,^2009^; Shulman and Guberman, 2007; for ASD research see Naigles et al., 2011). However, it is also important to demonstrate productivity in actual production and that is the aim of the current paper.

^2^Fusaroli, R., Weed, E., Fein, D., Naigles, L. (2020). Caregiver linguistic alignment to autistic and typically developing children: a natural language processing approach illuminates the interactive components of language Development. Unpublished manuscript.

^3^We first attempted to use random effects to account for the variance from the participants in a linear mixed-effects modeling approach. However, these random effects led to overfitting in the model in which the estimate of variance became extremely small (i.e., e-16). Therefore, the random effects by participant were dropped from the model, resulting in a linear modeling approach. The same issue emerged for all other models, so random effects are not included in any models.
